# Associations between oral health-related impacts and rate of weight gain after extraction of pulpally involved teeth in underweight preschool Filipino children

**DOI:** 10.1186/1471-2458-13-533

**Published:** 2013-06-03

**Authors:** Denise Duijster, Aubrey Sheiham, Martin H Hobdell, Gina Itchon, Bella Monse

**Affiliations:** 1 Department of Preventive Dentistry, Academic Centre for Dentistry Amsterdam, Gustav Mahlerlaan 3004, 1081LA, Amsterdam, The Netherlands; 2 Department of Epidemiology and Public Health, University College London, Torrington Place 1-19, London WC1E 6BT, UK; 3 Department of Preventive and Community Medicine, Dr. Jose P. Rizal College of Medicine, Xavier University, Ateneo de Cagayan, 9000, Cagayan de Oro City, Philippines; 4 Gesellschaft für Internationale Zusammenarbeit (GIZ), Leviste cor Rufino Street, Makati City, Metro Manila, Philippines

**Keywords:** Dental caries, Dental decay, Tooth extraction, Oral impacts, Sleeping, Eating, Dental pain, Underweight, Weight gain, Growth

## Abstract

**Background:**

Severe dental caries in young children is associated with underweight and failure to thrive. One possible mechanism for severe caries affecting growth is that the resulting pain and discomfort influences sleeping and eating, and that affects growth and weight. The objective of this study was to assess whether rate of weight gain after extraction of severely decayed teeth in underweight preschool Filipino children was related to reductions in oral health-related impacts and dental pain from severe dental caries affecting eating and sleeping.

**Methods:**

Data are from the Weight Gain Study, a stepped wedge cluster randomized clinical trial where underweight Filipino children with severe dental decay had their pulpally involved teeth extracted. Day care centers were randomly divided into two groups; A and B. Group A children received treatment first and Group B children were treated four months after Group A. Clinical oral examinations used WHO criteria and the pufa-index. Self-reported oral health-related impacts and anthropometric measurements were collected for both groups at baseline, four months after treatment of Group A children and four months after treatment of Group B children. Weight-for-age *z*-scores were calculated using 2006 and 2007 WHO standards. Data were converted to a one-group pre-test post-test study design, where all children received treatment. Associations between changes in oral health-related impacts and weight-for-age *z*-scores after dental treatment were assessed.

**Results:**

Data on 145 children (mean age 61.4 months) were analyzed. There was a significant association between oral health-related impacts and rate of weight gain after extraction of pulpally involved teeth (p=0.02). Children free of impacts on sleeping related to having severely decayed teeth extracted gained significantly more weight compared to children who reported sleeping problems after dental treatment (p<0.01).

**Conclusions:**

After extraction of severely decayed teeth in underweight Filipino children, levels of oral health-related impacts were associated with rate of weight gain. Decreases in oral health impacts on sleeping appeared to be most strongly associated with weight gain.

**Trial registration:**

ISRCTN: ISRCTN90779069

## Background

Untreated severe dental caries in preschool children is very common in many low- and middle-income countries [1]. In the Philippines, caries levels are among the highest in South-East Asia. The 2006 Philippine National Oral Health Survey revealed that the mean number of decayed or missing teeth in 6-years-olds was 8.4 [2]. Children had on average 3.4 teeth where caries had progressed into the pulp.

Severe dental caries affects young children’s quality of life and well-being. Children with untreated early childhood caries (ECC) had significantly poorer oral health-related quality of life (OHRQoL) than those without ECC [3]. Dental rehabilitation resulted in significant improvements in children’s OHRQoL with significant reductions in dental pain, sleeping and eating problems, and their eating and appetite improved [4-7].

Poor oral health in children is associated with underweight and failure to thrive [8]. Children requiring multiple extractions of severely decayed teeth had significantly lower body weights than caries free children [9-11]. Similar results were observed in the Philippine National Oral Health Survey [12]. Alkarimi demonstrated that there is a graded inverse relationship between severe dental caries and weight, height and BMI in Saudi children [13]. Dental rehabilitation of underweight children with severe dental caries was associated with an increased rate of weight gain [14,15]. Extraction of pulpally involved primary teeth resulted in significant weight gain in underweight children participating in the Weight Gain Study (WGS), a cluster randomized clinical trial conducted in the Philippines [16].

There are several plausible mechanisms explaining why dental rehabilitation leads to an increased rate of weight gain. Severe dental decay can cause dental pain and that can negatively affect children’s ability to eat and sleep [4,5]. Impacts on eating may affect the quality and quantity of nutritious food consumed, resulting in inadequate caloric intake, while disturbed sleep due to dental pain can affect the secretion of growth hormones [17] or may cause excessive energy expenditure. Another theory is that dental inflammation from pulpally involved teeth suppresses growth through a metabolic pathway by reducing hemoglobin levels as a result of depressed erythrocyte production in the bone marrow [18]. However, none of these theories have been thoroughly tested. The objective of this study was to assess whether rate of weight gain after extraction of severely decayed teeth in underweight preschool Filipino children was related to reductions in oral health-related impacts and dental pain from severe dental caries affecting eating and sleeping.

## Methods

### Study design

**Figure 1:**
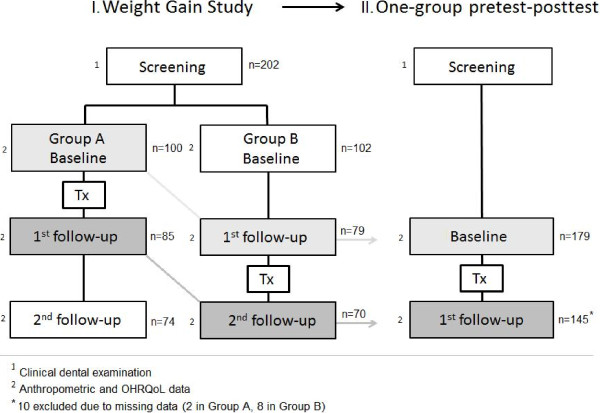
Study design.

The present study used data from the Weight Gain Study, which was a stepped wedge cluster randomized clinical trial [19,20]. In the WGS, thirteen day care centers in ten municipalities in the province of Misamis Oriental were randomly divided into two groups; intervention Group A (six day care centers) and waiting list control Group B (seven day care centers). Children from Group A (*n*=100) had all pulpally involved primary teeth extracted under local anesthesia [17]. Their other carious teeth were treated with silver-diamine-fluoride Arrest of Caries Technique (ACT) [21]. Children in Group B (*n*=102) were treated four months later in the same way as Group A children. Anthropometric measurements, data on oral health-related impacts and blood samples were collected at the same time for both groups at the beginning of the study, four months after treatment of Group A children and four months after treatment of Group B children (Figure 1, Design I).

For this study data from the WGS were converted into a one-group pretest-posttest study design where all children received treatment. Baseline data existed of the measurements of all children from Groups A and B, collected immediately before they received dental treatment. The follow-up data were measurements collected four months after the children were treated (Figure 1, Design II).

### Study population

Children in the WGS, aged between 48 and 68 months, were recruited from day care centers in municipalities in the province of Misamis Oriental, Northern Mindanao, Philippines. All children were underweight and had severe dental caries, with one or more pulpally involved primary teeth. Children were defined as underweight if their BMI was below the 5th percentile according to CDC Growth Charts. Children who tested positive for active tuberculosis infection at the screening stage were excluded, and referred for treatment. Mentally handicapped children were also excluded for ethical reasons. None of the included children had systemic medical conditions and/or infectious diseases, according to parental reports. All parents or caregivers signed an informed consent. Written ethical approval was obtained from the Ethics Commission of Xavier University, Cagayan do Oro City.

### Data collection

XPrior to group assignment, all children were orally screened. Socio-demographic data were collected through a parental questionnaire and a face-to-face interview with parents at baseline. At each stage of the trial, data collection involved the collection of anthropometric measurements, completion of an interview-administered OHRQoL-questionnaire and collection of blood samples. Blood samples were taken to assess the effect of extraction of severely decayed teeth on hemoglobin levels to explore a metabolic pathway between severe dental caries and body growth, which will be reported in a separate paper. This paper reports only on the data relating to weight gain and oral health-related impacts.

#### Oral screening

Clinical dental data were collected using standard WHO basic methods [22]. One trained and calibrated general dentist carried out all examinations. Children were examined outside the day care centers, using the daylight as direct light source. They were examined lying in supine position on the carers’ and examiner’s laps (‘knee to knee’ position). Caries was scored when the ball end CPI probe could penetrate the dental caries cavity. Caries experience was recorded using the dmft-index. In addition, the severity of oral conditions resulting from untreated severe dental decay was scored using the pufa-index [23]. The pufa-index records presence of severely decayed teeth with visible pulpal involvement (p), ulceration caused by dislocated tooth fragments (u), fistula (f) and abscess (a). The pufa-score per person is calculated in the same cumulative way as for the dmft-score and represents number of teeth meeting pufa diagnostic criteria. Children with a minimum of one pulpally involved tooth (pufa-score of at least 1) were included in the study.

#### Data on oral health-related impacts

Data on children’s oral health-related impacts were collected using the Scale of Oral Health Outcomes (SOHO-5); an interviewer-administered OHRQoL questionnaire developed for five-year old children [24]. Tsakos et al. demonstrated that the SOHO-5 is an internally consistent and valid questionnaire: SOHO-5 scores were significantly associated with different subjective oral health outcomes of 5-year old schoolchildren and scores discriminated between different clinical groups in relation to active caries lesions, pulp involvement and dental sepsis [24]. The SOHO-5 was translated into Visayan, the local language, and back translated into English. A pilot study showed that the questionnaire could be used in the Philippine setting. The SOHO-5 questionnaire contained ten questions; seven of these assessed oral health-related impacts, including eating difficulty, drinking difficulty, speaking difficulty, difficulty playing, avoiding smiling (due to appearance), avoiding smiling (due to pain), and difficulty sleeping, two questions measured current and past toothache and one question assessed children’s satisfaction with oral health. All questions were assessed using a 3-point scale with the answering options ‘no’ (coded 0), ‘a little’ (coded 1) and ‘a lot’ (coded 2). A cumulative SOHO-5-score was calculated for the seven items on oral health-related impacts, which reflects the overall presence of oral health-related impacts, ranging from 0 (free from impacts) to 14 (most severe possible score). Only the question assessing current toothache was used in the present study.

#### Anthropometric data

Weight and height was measured in duplicate by a trained nurse who was blinded as to which children had dental extractions first. She used the ‘WHO Anthropometric Indicators Measurements Guide’ [25]. The average of two readings was recorded. Weight was measured to the nearest 0.1 kg using portable hanging scales (Salter scale, UNICEF procurement), and calibrated after every five measurements. Standing height was measured to the nearest 0.1 cm using a stadiometer (‘Leicester’ Model, Children’s Growth Foundation, UK). Weight and height measurements were converted to *z*-scores, namely weight-for-age (WAZ), height-for-age (HAZ) and BMI-for-age (BAZ) with the lmsGrowth Excel add-in (Medical Research Council, 2008), using the 2006 [26] and 2007 [27] WHO Growth Standards. *Z*-scores allow comparison of an individual’s weight, height or BMI, adjusting for age and sex relative to a reference population, expressed in standard deviations (SD) from the reference mean.

### Statistical analysis

Statistical analysis was carried out using STATA 10 (Stata Corp, College Station, Texas, USA). A p-value of <0.05 was regarded as significant. All children with missing data due to loss to follow-up or unrecorded data were excluded from the analysis.

Oral health-related impacts and anthropometric measurements recorded before and after treatment were assessed using the paired *T*-test and the McNemar test. A series of simple linear regression analyses were performed to assess baseline associations between presence of oral health-related impacts and WAZ at baseline, and to assess the association between oral health-related impacts and weight gain after treatment. Weight gain was the outcome variable in the regression models, which was expressed as the difference in WAZ between before and after dental treatment. The explanatory variables referred to oral health-related impacts, which were analyzed one by one in simple linear regression models as the overall SOHO-5-score, eating impacts, sleeping impacts and dental pain. Oral health-related impacts were expressed in two ways; as changes in oral health-related impacts between before and after treatment, categorized as ‘improvement’, ‘no difference’ or ‘deterioration’, and as the prevalence of oral health-related impacts after treatment (dichotomized as ‘present’ or ‘absent’). The categories ‘deterioration’ and ‘present’ were used as reference values.

Then the number of teeth with dentally-related infections (pufa-score), WAZ at baseline and the time interval between treatment and follow-up were entered in the regression analyses to test whether these covariates moderated the association between oral health-related impacts and weight gain.

## Results

Two hundred and two children participated in the WGS (100 children in Group A and 102 in Group B). Eighty one percent of them (164 children; 85 in Group A and 79 in Group B) were reassessed at the first follow-up stage four months later and 71.3% (144 children; 74 in Group A and 70 in Group B) completed all stages of the study. The frequent migration of families was the main reason for loss to follow-up.

For this study, data on 145 children (83 from Group A and 62 from Group B) were analyzed (71.8%), after excluding all children with missing data due to drop-out or unrecorded data (Figure 1, Design II).

### Characteristics at baseline and follow-up

The mean age of the children at baseline was 61.4±5.1 months (Table 1). There were significantly more girls (62.1%) than boys (37.9%) (p=0.02). The mean dmft of the children was 10.9±4.4. All children had at least one pulpally infected tooth at baseline, with an average pufa-score of 2.3±1.6. They had a mean of 2.0±1.7 teeth with pulp involvement (88.7%), 0.2±0.5 teeth with a fistula (9.1%) and 0.05±0.02 teeth with an abscess (2.1%). In this study on average 2.2±1.1 teeth were extracted per child, ranging from one to nine extractions. The mean time interval between the extraction of pulpally involved teeth and the follow-up was 4.5±0.8 months.

**Table 1:** Characteristics of the study population at baseline

***Characteristics***	***n***	***%***	
Male	55	37.9	
Female	90	62.1	
	*Mean ± sd*	*Range*	*Median*
Age (months)	61.4 ± 5.1	48 - 71	61.8
dmft-score	10.9 ± 4.4	2 - 20	9
Pufa-score*	2.3 ± 1.6	1 - 13	2
No. of extractions	2.2 ± 1.1	1 - 6	2
Monthly income (US$)**	99 ± 56	12 - 331	83
Time interval between baseline and first follow-up (months)	4.5 ± 0.8	2.9 – 6.4	4.5

*9 missing observations, **22 missing observations.

All children were underweight at baseline. The average WAZ, HAZ and BAZ *z*-scores were 2.14±0.6, 1.58±0.8 and 1.67±0.5 SD, respectively, below the mean of the reference population (Table 2). All children gained a significant amount of weight and height between baseline and follow-up four months after surgical tooth extraction. The average WAZ and BAZ significantly increased after dental treatment, while HAZ significantly decreased. The increase in WAZ and BAZ from a negative value to a less negative value implies that children were closer to the weight-for-age and BMI-for-age of the reference population of the same age and sex after treatment, which means that children had an increased rate of weight gain. The decrease in HAZ implies that children gained less height than would be expected in four months based on their age and sex relative to the reference population. Seventy-eight percent of the children increased their WAZ after treatment, while the WAZ of 28% of the children decreased (results not shown in a table).

**Table 2:** Anthropometric measurements before and after treatment

	**Before treatment**		**After treatment**		
	***Mean***	***95% CI***	***Mean***	***95% CI***	p-value*
Weight (kg)	13.9	(13.7, 14.1)	15.1	(14.8, 15.4)	<0.001
Height (cm)	102.7	(102.0, 103.4)	104.3	(103.5, 105.1)	<0.001
BMI (kg/m^2^)	13.2	(13.1, 13.3)	13.8	(13.7, 13.9)	<0.001
WAZ (SD)^•^	−2.14	(−2.24, -2.04)	−1.88	(−2.01, -1.75)	<0.001
HAZ (SD) ^•^	−1.58	(−1.72, -1.44)	−1.67	(−1.82, -1.52)	<0.001
BAZ (SD)^•^	−1.67	(−1.75, -1.52)	−1.15	(−1.27, -1.03)	<0.001

*Paired *T*-test.

^•^*WAZ* = weight-for-age, *HAZ* = height-for-age, *BAZ* = *BMI*-for-age (*Z*-scores).

Before treatment the average SOHO-5-score was 4.1±3.4 (Table 3); 120 children (82.8%) reported at least one impact. Four months after treatment the average SOHO-5-score significantly decreased to 1.4±2.9, with only 40 children (27.6%) still reporting an impact. There was a significant reduction after surgical tooth extraction in the percentage of children having eating impacts, sleeping impacts and dental pain. The majority of children experienced less oral health-related impacts after dental treatment. However, 15.9%, 9.7%, 11.0% and 5.5% children reported a deterioration in SOHO-5-scores relating to eating impacts, sleeping impacts and dental pain, respectively.

**Table 3:** Oral health-related impacts before and after treatment

	**Before treatment**		**After treatment**		
	***Mean***	***95% CI***	***Mean***	***95% CI***	p-value*
Overall SOHO-5 score	4.1	(3.5, 4.7)	1.4	(0.9, 1.9)	<0.001
	n	%	n	*%*	p-value**
Eating impact					<0.001
*Yes*	104	71.7	31	21.4	
*No*	41	28.3	114	78.6	
Sleeping impact					<0.001
*Yes*	86	59.3	29	20.0	
*No*	59	40.7	116	80.0	
Dental pain					<0.001
*Yes*	36	24.8	12	8.3	
*No*	109	75.2	133	91.7	

*Paired *T*-test, **McNemar test.

### Baseline associations between oral health-related impacts and weight

An increase of one in SOHO-5-score was significantly associated with a 0.03SD (0.00, 0.06) lower WAZ at baseline (Table 4). Children reporting eating impacts at baseline had significantly lower body weights than children who were free of eating impacts, yet no significant associations were found for sleeping impacts and for presence of oral pain with WAZ at baseline (Table 4).

**Table 4:** **The association between oral health-related impacts and WAZ**^• ^**at baseline**

	**WAZ*β-coefficient***	***95% CI***	p-value*
SOHO-5 score (continuous)^i^	0.03	(0.00, 0.06)	0.05
Eating impact^ii^	0.26	(0.04, 0.48)	0.02
Sleeping impact^ii^	0.16	(−0.05, 0.36)	0.14
Dental pain^ii^	0.04	(−0.20, 0.27)	0.75

*Simple linear regression.

^i^SOHO-5 score at baseline ii ‘absent’ regressed against ‘present’ (reference).

^•^WAZ = weight-for-age (*Z*-score).

### Associations between oral health-related impacts and weight gain after treatment

Children with improved SOHO-5 scores after tooth extraction gained significantly more weight compared to children who deteriorated in SOHO-5 scores after treatment (Table 5). Having a lower SOHO-5 score after treatment was also associated with an increased rate of weight gain, compared to children with higher SOHO-5 scores after treatment (Table 6).

**Table 5:** The association between changes in oral health-related impacts between before and after treatment and weight gain after treatment

		**WAZ-difference**^**•**^***β-coefficient***	***95% CI***	p-value*
SOHO-5-score (continuous)^i^		0.01	(−0.01, 0.04)	0.23
SOHO-5-score				
	*No difference*^ii^	0.21	(−0.06, 0.48)	0.13
	*Improvement*^ii^	0.30	(0.10, 0.50)	0.003
Eating impact				
	*No difference*^ii^	0.04	(−0.23, 0.32)	0.75
	*Improvement*^ii^	0.11	(−0.14, 0.37)	0.39
Sleeping impact				
	*No difference*^ii^	0.36	(0.12, 0.61)	0.004
	*Improvement*^ii^	0.31	(0.07, 0.55)	0.01
Dental pain				
	*No difference*^ii^	0.24	(−0.07, 0.55)	0.13
	*Improvement*^ii^	0.19	(−0.14, 0.52)	0.25

*Simple linear regression.

^i^Difference in SOHO-5 score between before and after treatment.

^ii^‘improvement’ and ‘no difference’ regressed against ‘deterioration’ (reference).

^•^Difference in weight-for-age (*Z*-score) between before and after treatment.

**Table 6:** The association between oral health-related impacts after treatment (presence or absence) and weight gain after treatment

	**WAZ-difference**^**•**^***β-coefficient***	***95% CI***	p-value*
SOHO-5-score (continuous)^i^	0.04	(0.01, 0.05)	0.04
Eating impact^ii^	0.16	(−0.02, 0.33)	0.09
Sleeping impacts^ii^	0.26	(0.08, 0.44)	0.006
Dental pain^ii^	0.17	(−0.10, 0.44)	0.21

*Simple linear regression.

^i^‘SOHO-5-score after treatment.

^ii^‘absent’ regressed against ‘present’ (reference).

^•^Difference in weight-for-age (*Z*-score) between before and after treatment.

Simple linear regression analyses of the influence of individual impacts on weight gain showed that children who improved or reported no difference in their ability to sleep, gained significantly more weight than children whose sleeping ability deteriorated (Table 5). A similar association was found between the presence or absence of sleeping impacts after treatment and weight gain; children free from sleeping impacts after tooth extraction had a significant increased rate of weight gain compared to children who reported sleeping impacts after extractions (Table 6). No significant associations were found between weight gain and changes in eating impacts and changes in dental pain experience after treatment.

The number of teeth with dental infections, WAZ at baseline and the time interval between the extraction of severely decayed teeth and follow-up was not significantly related to rate of weight gain (Table 7). Therefore it was not necessary to adjust the association between oral health-related impacts and weight gain for these covariates in multiple regression models.

**Table 7:** The association between covariates and weight gain

***Covariate***	**WAZ-difference**^**•**^***β-coefficient***	***95% CI***	p-value*
pufa-score	0.02	(−0.03, 0.07)	0.48
WAZ at baseline	0.06	(−0.06, 0.18)	0.35
Time interval	0.05	(−0.04, 0.13)	0.30

*Simple linear regression.

^•^Difference in weight-for-age (*Z*-score) between before and after treatment.

## Discussion

Findings of this intervention study indicate that there was a statistically significant association between self-reported oral health-related impacts and the rate of weight gain after extraction of severely decayed teeth in preschool Filipino children. At baseline, eating impacts were related to reduced body weight. However, improvement in eating ability, or being free from eating impacts after surgical tooth extraction, was not associated with weight gain. On the other hand, reductions in the impacts on sleeping had a consistent significant association with increased rate of weight gain, suggesting that the impact on sleeping might be an important factor in the relationship between extraction of severely decayed teeth and weight gain.

Impacts on sleeping can play an important role in growth retardation due to disturbance in production of growth hormones resulting from lack of sleep [17]. Based on theories of such a mechanism, it is expected that sleeping would have a stronger impact on height than on weight. However, in this study the height-for-age significantly decreased after dental treatment, suggesting that children grew less in height in four months than would be expected based on their age and sex, relative to a reference population. Height, however, takes longer to change than weight. The process of growth is described as saltation and stasis, whereby infant growth follows a series of rapid growth spurts (saltation), separated by periods of stasis [28,29]. This indicates that children first put on weight before growing in height. This complex process of catch-up growth and the short timespan between dental extractions and follow-up in this study may explain why height did not increase. Height measurements were not regularly monitored. That, and the short time interval between baseline and follow-up that was necessary for ethical reasons, are reasons why it was not possible to rigorously assess the association between sleeping impacts and height gain after dental treatment.

Dental pain was hypothesized to be the intermediate factor for the associations between impacts on eating and sleeping and weight gain. However, there was no association between oral pain and body weight. That may be related to how dental pain was measured. Dental pain was assessed as ‘do your teeth hurt now?’. Since dental decay leads mainly to dental pain that is not permanently present, not all children with severe dental decay reported having dental pain at baseline. The number of children with dental pain at baseline and after dental treatment (n=36 and n=12, respectively) may have been too small to demonstrate a statistically significant association between dental pain and weight gain.

Oral health-related impacts were assessed in two ways: as changes in impacts between before and after treatment (‘improvement’, ‘no difference’ or ‘deterioration’) and as the prevalence of impacts after treatment (‘present’ or ‘absent’). The findings of this study suggest that the absence of oral health-related impacts after dental treatment is a stronger, and probably more accurate predictor of weight gain, compared to assessing a decrease in reported impacts between before and after treatment. Children who improved from having ‘a lot’ of impacts at baseline to ‘a little’ after treatment were still experiencing problems, while children who did not report a difference in impacts could have been free of impacts at both stages of the study.

A notable finding was that in some children weight-for-age decreased after dental treatment. This may be related to other factors, such as dietary factors, poor environments and other social and medical factors that confounded the association between severe dental decay and weight gain. These factors are more likely to have a stronger influence on weight gain than dental treatment. For example, the prevalence of soil-transmitted helminths in under-5-year-old children in the Philippines ranged from 49% to 93% [30]. Worm infestation has a negative impact on nutrition. Other medical factors, such as anemia and infectious diseases including malaria and diarrhea, can negatively influence weight gain [31]. Most children in this study were from very deprived municipalities, where access to food was the highest priority. This could contribute to parental stress, which is related to a child’s failure to thrive [31]. The aforementioned factors were not assessed in this study, and may have influenced the rate of weight gain and its association with oral health-related impacts.

Other factors that were hypothesized to moderate the association were number of pulpally infected teeth, weight-for-age at baseline and the time interval between treatment and follow-up. It was presumed that children with several infected teeth would benefit more from treatment, compared to children with only one tooth affected. Weight at baseline was hypothesized to be an important moderator, as there is a tendency for children who are taller, to grow slightly less than smaller children, a ‘regression to the mean’ [32]. The variation in time intervals between dental treatment and follow-up may have influenced the time for children to grow after tooth extractions. None of the abovementioned assumptions were supported by results of this study. Whether the relationship between the abovementioned covariates and weight gain are truly non-existent or if the non-significant findings resulted from the small sample size and effects of other medical, social and environmental factors, should be further investigated.

One of the main strengths of this study was that the data were derived from a stepped wedge cluster randomized clinical trial, which demonstrated the benefit on weight gain of dental extractions. For this study, data were converted to a prospective cohort design, allowing assessment of the relationship between oral health-related impacts and rate of weight gain over time. Some potential limitations of the study should however be taken into account. A fair number of children were lost to follow-up, which may have affected the results as children who dropped out may have responded differently to dental treatment than those children who completed all stages of the study. The unequal distribution of gender in the study sample may also have influenced the results. Although a validated questionnaire was used to collect information on oral health-related impacts, there may have been bias in children’s self-reports. The relatively small number of children in the study may have resulted in some weak associations between oral health-related impacts and weight gain and the time interval between surgical tooth extraction and follow-up may have been too short for children to gain much weight and height following treatment. A cause-effect relationship cannot be deduced from the one-group pretest-posttest design.

More research is needed to further explore the causal mechanisms between severe dental decay and body growth in young children. Studies should also investigate the metabolic pathways and incorporate parameters related to general health, psychosocial relationships and environmental factors, as body growth is influenced by numerous factors. The time interval between dental treatment and follow-up should be longer and anthropometric measurements should be regularly monitored to investigate the relationships between severe dental decay and height.

## Conclusions

This study shows that after extraction of severely decayed teeth in underweight Filipino children, levels of oral health-related impacts were associated with rate of weight gain. Decreases in oral health impacts on sleeping after tooth extraction appeared to be most strongly associated with weight gain.

Despite the growing recognition that a common condition, such as untreated severe dental decay affects children’s growth and well-being, investment in improving oral health is still neglected in many low- and middle-income countries. If the evidence of an impact of oral health on body constitution can be further established by understanding its underlying causal mechanisms, then emphasis on prevention of dental decay and the provision of access to basic oral care needs to be one of the priorities of integrated health promotion programs to enhance growth and well-being of millions of underweight children.

## Abbreviations

ACT: Arrest of caries technique; BAZ: BMI-for-age *z*-score; BMI: Body mass index; CPI: Community periodontal index; dmft: decayed, missing and filled teeth; ECC: Early childhood caries; HAZ: Height-for-age *z*-score; OHRQoL: Oral health related quality of life; pufa: Presence of severely decayed teeth with visible pulpal involvement, ulceration caused by dislocated tooth fragments, fistula, abscess; SD: Standard deviation; SOHO-5: Scale of Oral Health Outcomes for 5-year-old children; WAZ: Weight-for-age *z*-score; WGS: Weight gain study; WHO: World Health Organization.

## Competing interests

The authors declare that they have no competing interests.

## Authors’ contributions

Conception and study protocol (BM, AS, MHH), adaption of the questionnaires to the local context (GI), study implementation and data collection (BM), data analysis (DD), interpretation of findings (DD, AS, MHH, GI, BM), drafting of the initial manuscript (DD), revision of the manuscript (DD, AS, MHH, GI, BM), agreed to the final version of the manuscript (DD, AS, MHH, GI, BM).

## Pre-publication history

The pre-publication history for this paper can be accessed here:

http://www.biomedcentral.com/1471-2458/13/533/prepub
